# Leucorrheal Discharges; Their Causation and Treatment

**Published:** 1903-12

**Authors:** Clarence G. Clark

**Affiliations:** New York


### Leucorrheal Discharges; their Causation and Treatment.

By CLARENCE G. CLARK, M. D., New York.

THE vagina is seldom, if ever, the seat of idiopathic inflammation. Vaginitis is always either due to some specific cause or is secondary to some contiguous inflammation. Vaginitis may be classified according to duration as acute or chronic; to causation, as erysipelatous, diphtheritic, gonorrheal, and traumatic. The inflammation may also be general or circumscribed, but is usually the former. In this as in all other forms of inflammation there are present the cardinal symptoms of rubor, calor and dolor, or redness, heat and pain. The pathology differs slightly in the various forms. In general there is congestion, transudation of serum, premature exfoliation of epithelium and in well defined cases, the formation of pus. The vaginal membrane resembles more closely the structure of the skin and, therefore, vaginitis is more like dermatitis in character and occurs either as erythematous, purulent or exudative, but never as purely catarrhal. Only in the advanced cases of leucorrhea is pus found in any quantity, the disease progressing rapidly from a simple congestion with a thin serous discharge to an exfoliative vaginitis with copious discharge of serum, mixed with epithelial cells. Only in the gonorrheal forms is pus found from the start.

Simple vaginitis is common especially in women in delicate health and of strumous diathesis. This form is not as marked as those due to some contiguous inflammation. Metritis is almost always accompanied by a leucorrheal discharge. Subacute and chronic inflammation of the vagina may be caused by an inflammation in the immediate neighborhood of the canal, as dysentery or erysipelatous inflammation of the vulva or anus. The discharge of a simple vaginitis differs from that of a vulvitis in being less tenacious. Microscopical examination will aid in the diagnosis greatly. Only in the gonorrheal, erysipelatous, and diphtheritic forms are distinct microörganisms found.

Among the symptoms of the simple form may be mentioned first a feeling of heat and fulness in the vagina. In severe cases vesical cases, vesical and rectal tenesmus are frequent. Often there are constitutional symptoms, fever and loss of appetite. If great cleanliness be not observed, the discharge decomposes and gives rise to an offensive odor. In subacute and chronic forms the symptoms are same in character, but less in degree.

*Treatment.*—Under this head I will only discuss the treatment of the simple leucorrheal discharges which, if taken at their outset, may be easily controlled and more serious complications prevented. Before the treatment is started the cause should be discovered and treated. For the discharge itself treatment is purely local. Medicinal applications may be applied in one of two ways,—as solutions used in douches or on tampons, or in powder form. I have found a combination of these to be the best, using both liquid medicinal agents and powders and employing both tampon and douche. In both tamponning and douching I have found that glycothymoline gives prompt results. Various powders may be used. In subacute and chronic cases I employ markasol (bismuth borophenate). The treatment is carried out as follows: first, the entire canal is cleansed with a douche of glycothymoline and warm water in proportion of 1 ounce to the quart. Next, the canal is thoroughly dusted with the powder desired and a tampon of borated cotton inserted. This is removed at the expiration of 24 hours and the canal is again cleansed. I generally increase the strength of the douche from day to day. In cases where the discharge is especially profuse and seropurulent in character, the powder is not used at first, but the canal is douched twice daily and tampons saturated in glycothymoline inserted. For this I use one part of the medicine to four of water.

To further illustrate, I will quote two cases, recently treated:

Case I. Miss M. F., aged 21; presented herself August 25, 1903, complaining of discharge from vagina, which has been gradually getting worse. She described a sensation of fulness and heat in vagina and burning on urination. Examination—Patient in poor general health, and with indifferent appetite; menstruation irregular; complexion sallow and anemic. Vagina sensitive to touch and walls covered with serous discharge. Labia slightly excoriated. Under the microscope discharge is composed chiefly of epithelial cells and few leucocytes.

*Treatment.*—Douched patient with glycothymoline and water 1-20. After thoroughly cleansing the vagina of all discharge, it was dusted with markasol and a tampon of borated cotton was inserted. Blaud's pills were administered. This treatment was continued for ten days, at which time the discharge was greatly diminished, I then discontinued the powder and prescribed douches of glycothymoline and water in proportion of 1 ounce to the quart, to be used twice daily. After about three weeks the patient was entirely free of discharge, menstruation was regular and general health much improved.

Case II. Mrs. H., April 23, 1903, complained of painful menstruation, with a copious discharge between menstrual periods. Married five years, one child, two years old. Forceps delivery. Had no trouble prior to birth of the child. Vagina was filled with mucopurulent discharge. Cervix lacerated, uterus enlarged and retroverted. Diagnosis: laceration of cervix, causing subinvolution and retroversion of womb and leucorrheal discharge.

*Treatment.*—Repaired cervix May 10, 1903. Patient in bed ten days. After recovery from the operation the discharge diminished and became more watery in character. Then began douching with glycothymoline in 1-20 solution. Patient had next menstrual period June 1, with very little pain, and after this subsided the discharge was very slight. The douching was continued twice daily for another month, when the discharge had vanished entirely and the menstrual period came without pain.

This case furnished a good example of the persistent discharge which may be produced from a laceration of the cervix. It is absolutely useless to attempt to relieve this character of discharge without first repairing the cause. In conclusion, would state that in occasional chronic cases, I have used glycothymoline pure, in an atomiser with satisfactory results.

---

### CHRONIC RHEUMATISM IN CACHEXIA.

℞ Potass. iodid.......................................... ½ oz.

Ol. morrhuæ.......................................... 3 ozs.

Tongaline.......................................... 3 ozs.

M. Sig. A teaspoonful every four hours.

